# Supplementary figures and images for: The Toxoplasma oxygen-sensing protein, TgPhyA, is required for resistance to interferon gamma-mediated nutritional immunity in mice
Source: PLoS Biol. 2024 Jun 10;22(6):e3002690. doi: 10.1371/journal.pbio.3002690 (PMC11192375; doi:10.1371/journal.pbio.3002690)

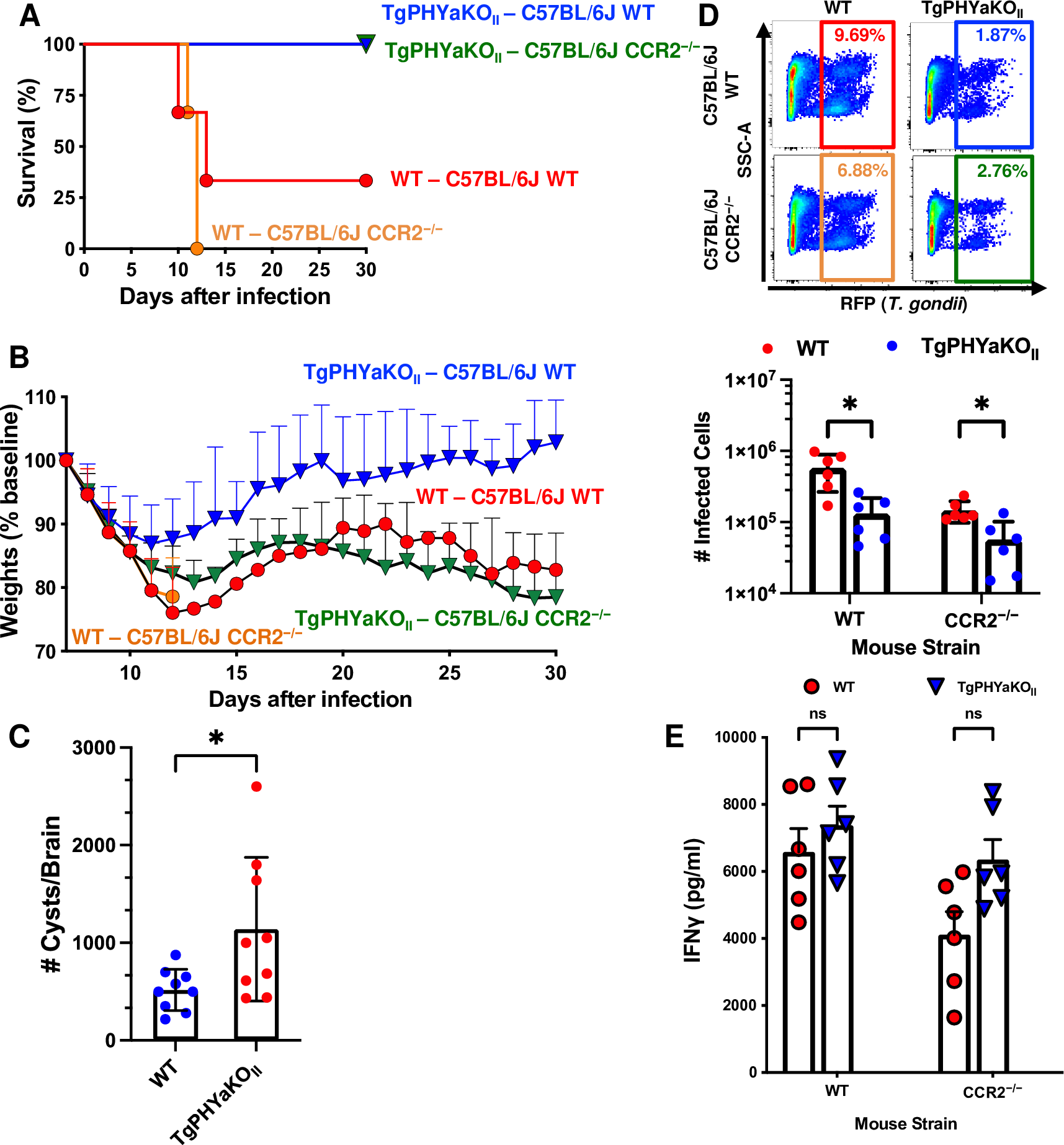

Supplement: S1 Fig — C57BL/6J WT and C57BL/6J CCR2-/- mice were orally infected by gavage with 50 cysts of ME49 WT or TgPHYaKOII parasites. (A) Kaplan–Meier curve showing survival of C57BL/6J WT and C57BL/6J CCR2-/- mice. Cumulative data from 3 independent experiments (n = 9 total for each strain). (B) Percent of weight loss of infected mice compared with initial weight before infection is plotted as mean ± SD from 3 independent experiments. (C) Cyst burdens in surviving mice 30 days after infection is plotted as mean ± SD. * P < 0.05 Student’s t test. (D) C57BL/6J WT and C57BL/6J CCR2-/- mice were gavage infected with 50 cysts of ME49 WT or TgPHYaKOII parasites. After 7 days infection, mice were euthanized and cells from small intestine lamina propria were processed and total number of infected cells was analyzed by flow cytometry as previously described (mean ± SEM, n = 6, pooled from 3 independent experiments). * P < 0.05 Student’s t test. (E) IFN-γ levels in serum of C57BL/6J WT, IFN-γ -/-, and CCR2-/- mice 7 days after oral infection with 50 cysts of ME49 WT or TgPHYaKOII parasites was quantified by an ELISA (mean ± SEM, n = 2–6, pooled from 3 independent experiments). The data underlying the graphs in this figure can be found in S2 Table. (TIF) [file pbio.3002690.s001.tif]

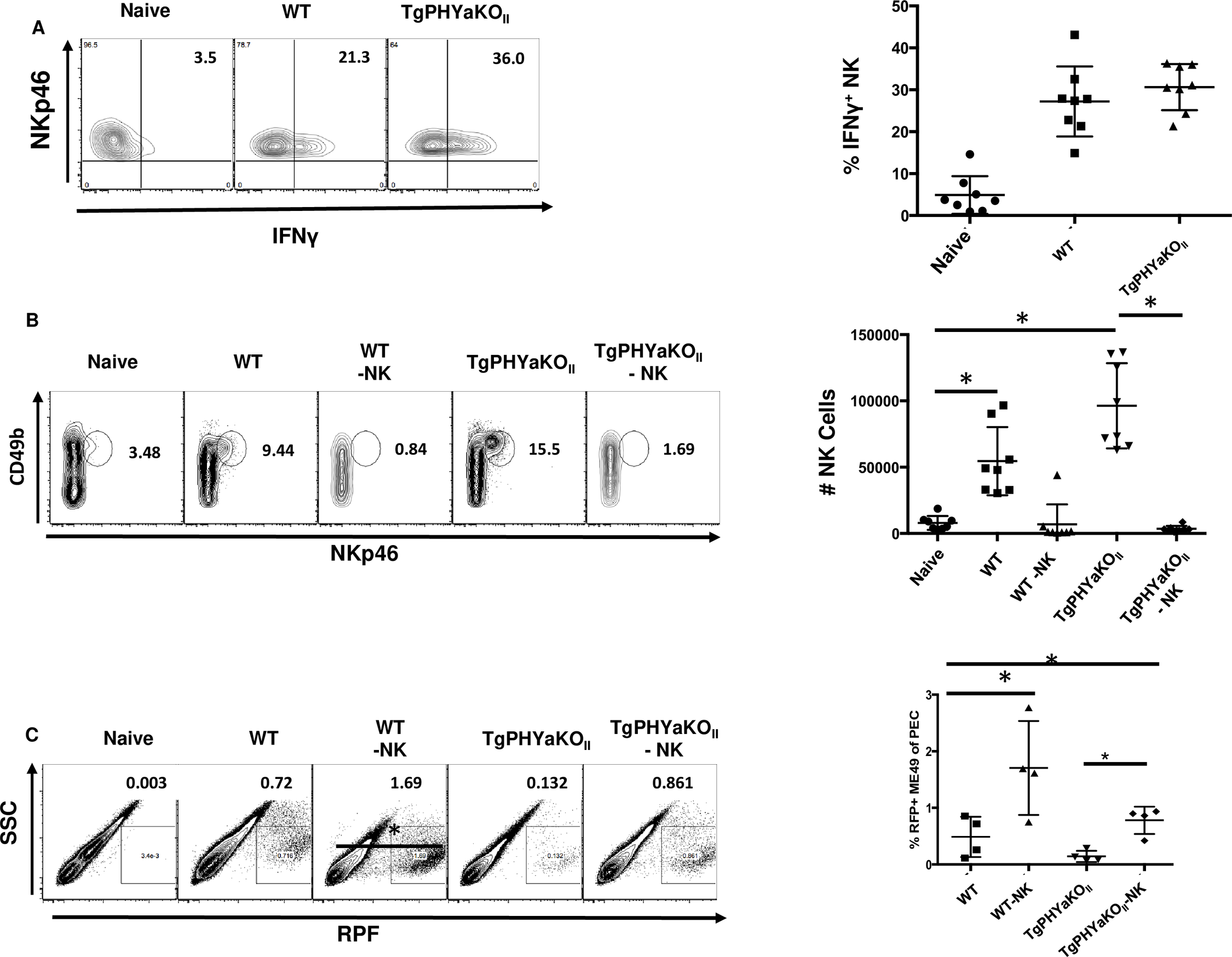

Supplement: S2 Fig — (A) Mice were mock-infected or infected IP with the indicated strains. After 7 days, peritoneal exudate was harvested and IFNγ expression in NK cells was determined by FACS. Shown are representative FACS plots and graphs are of 2 independent experiments with 2 mice per experiment. (B and C) Mice treated with anti-NK1.1 (or Mouse IgG2A) were infected with the indicated strains, and 7 days later peritoneal exudate was harvested and analyzed to assess NK cell depletion (B) or parasite burdens (C). The data underlying the graphs in this figure can be found in S2 Table. (TIF) [file pbio.3002690.s002.tif]

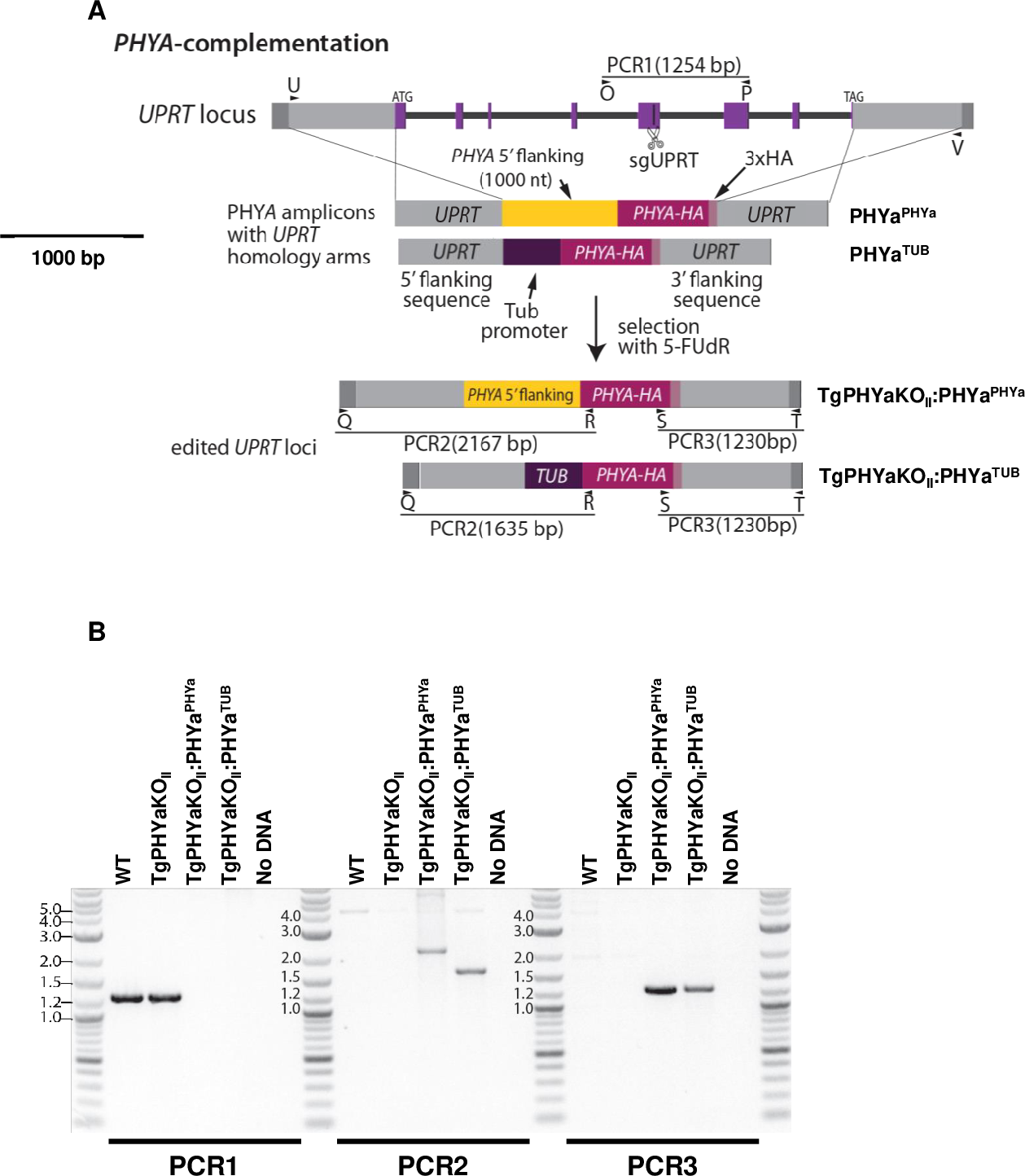

Supplement: S3 Fig — (A) Scheme for using CRISPR to target TgPHYa expression constructs to the UPRT locus. (B) Genomic DNA from the indicated strains was analyzed by PCR using the primers depicted in (A). Original blots can be found in S1 Raw Images. (TIF) [file pbio.3002690.s003.tif]

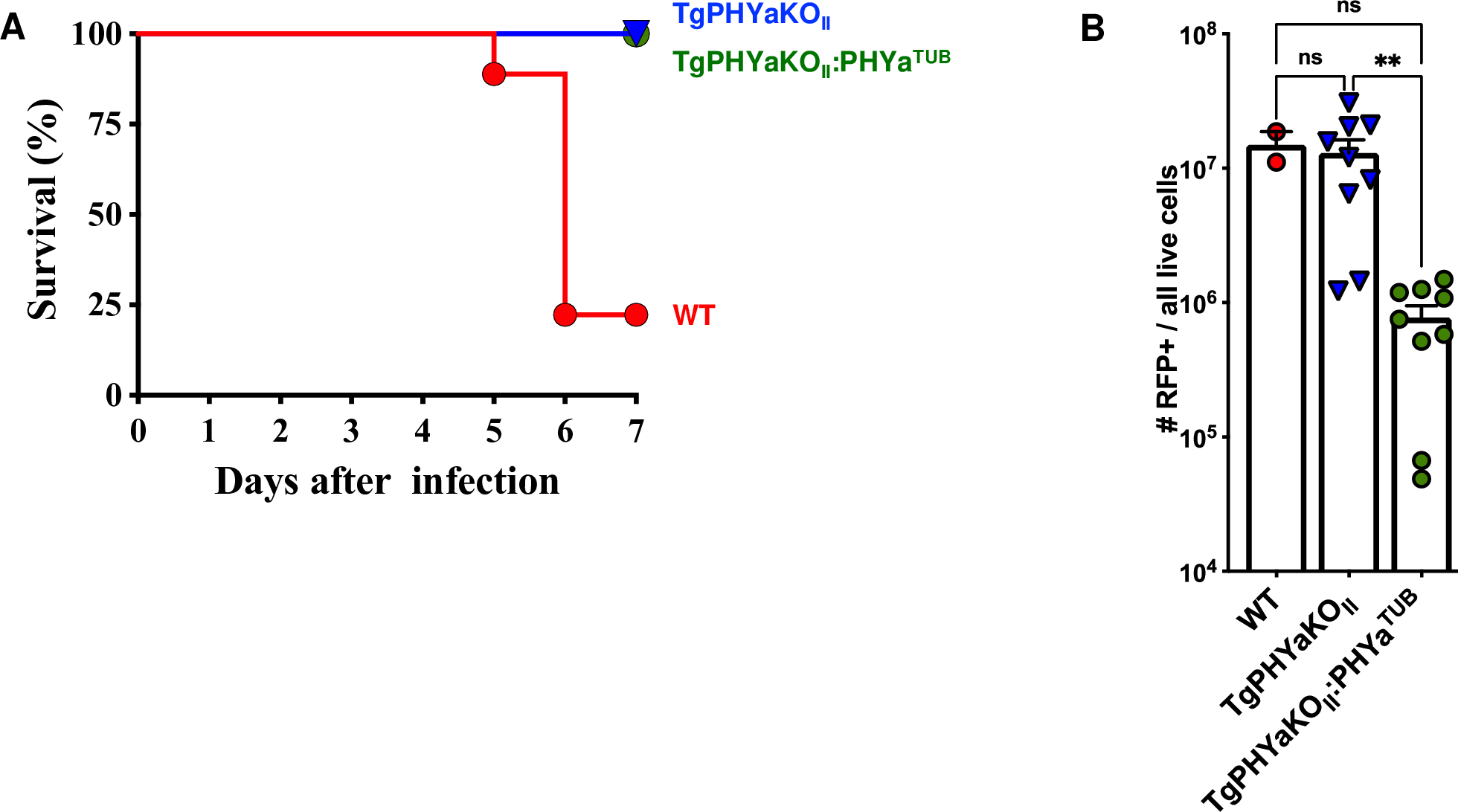

Supplement: S4 Fig — C57BL/6J WT mice were intraperitoneally infected with 106 tachyzoites of ME49 WT, TgPHYaKOII, or TgPHYaKOII:PHYaTUB parasites. (A) Kaplan–Meier curve showing survival of infected mice. Cumulative data from 3 independent experiments (n = 9 total for each strain). (B) Total number of infected cells was determined by flow cytometry. Plots from single-cells suspension of intraperitoneal cavity are gated on live cells. Analysis from 1 experiment (mean ± SEM, n = 6, pooled from 3 independent experiments; ***P < 0.001, using unpaired Student t test). The data underlying the graphs in this figure can be found in S2 Table. (TIF) [file pbio.3002690.s004.tif]

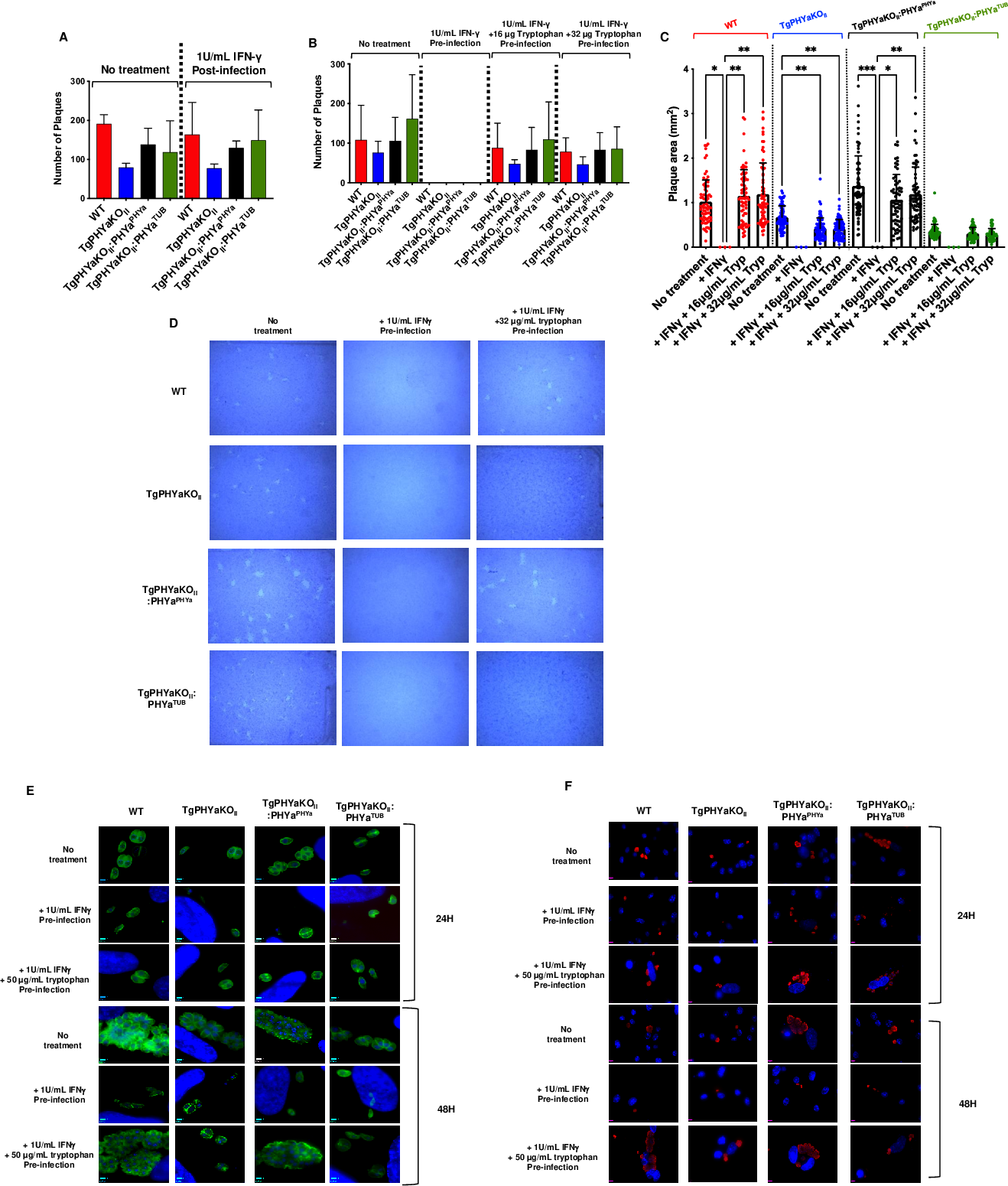

Supplement: S5 Fig — (A) Toxoplasma-infected HFFs were mock or IFNγ treated 24 hpi and 12 days later the monolayers were fixed and numbers and sizes of plaques determined (mean ± SEM, pooled from 3 independent experiments; ns = not significant, **P < 0.01 using a multiple comparison two-way ANOVA test). (B) HFFs were pretreated with IFNγ (1 U/ml) for 24 h and then infected with tachyzoites of the indicated strain. The cells were grown for 12 days in the presence of increasing tryptophan concentrations and then the monolayers were fixed and numbers of plaques enumerated. (C) HFFs were pretreated with IFNγ (1 U/ml) for 24 h and then infected with tachyzoites of the indicated strain. The cells were grown for 12 days in the presence of increasing tryptophan concentrations and then the monolayers were fixed and plaque sizes enumerated. Shown are the means ± SEM, pooled from 3 independent experiments; ns = not significant, *P < 0.05, **P < 0.01, using a multiple comparison two-way ANOVA test. (D) Representative images of plaques from S5C Fig. (E and F) Representative images of fields used for quantification in Fig 8C and 8D, respectively. The data underlying the graphs in this figure can be found in S2 Table. (TIF) [file pbio.3002690.s005.tif]

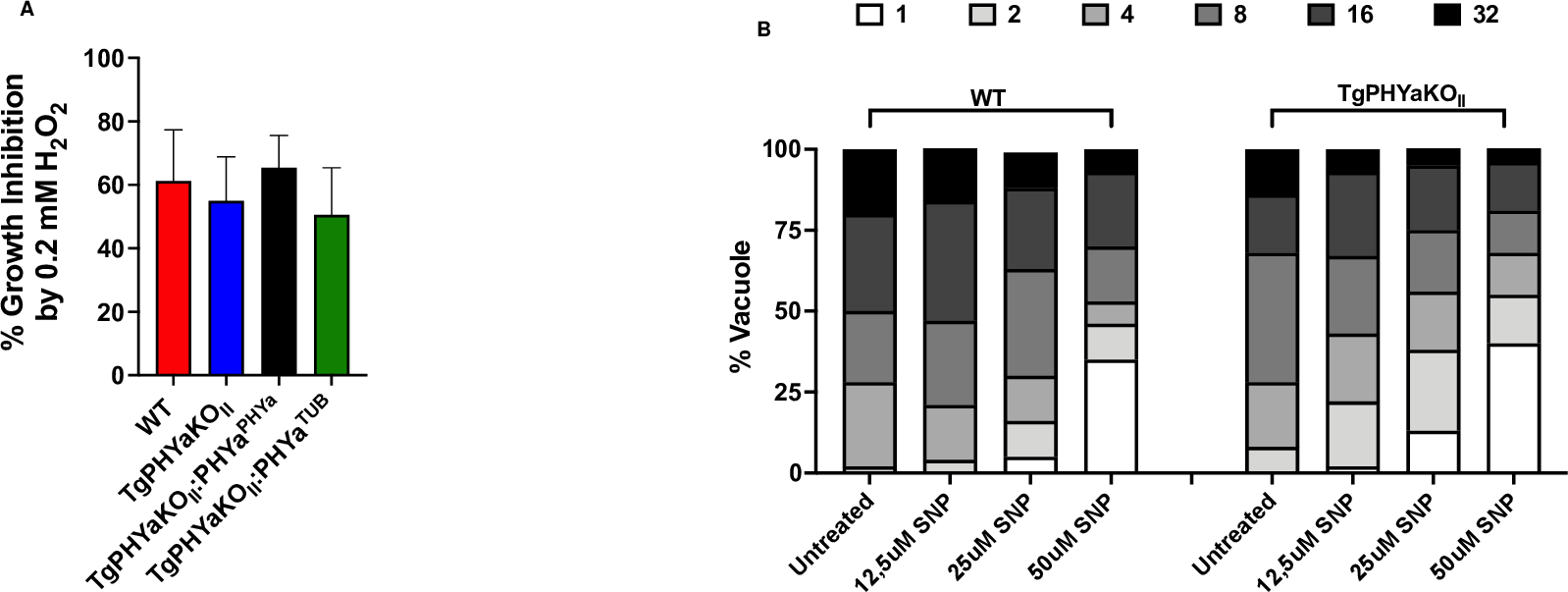

Supplement: S6 Fig — (A) Equal number of parasites were added to HFFs and grown in the presence of 0.2 mM H2O2. After 12 days, monolayers were fixed, numbers of plaques were counted, and results are expressed as the percentage of plaques formed relative to each strain grown without H2O2. (B) HFFs infected with tachyzoites of the indicated strain and then grown for 48 h in the presence of the indicated concentration of SNP. The monolayers were then fixed and numbers of parasites per vacuole determined by IFA staining. The data underlying the graphs in this figure can be found in S2 Table. (TIF) [file pbio.3002690.s006.tif]
