# Supplementary material for: The Toxoplasma oxygen-sensing protein, TgPhyA, is required for resistance to interferon gamma-mediated nutritional immunity in mice
Source: PLoS Biol. 2024 Jun 10;22(6):e3002690. doi: 10.1371/journal.pbio.3002690 (PMC11192375; doi:10.1371/journal.pbio.3002690)
Supplement: S1 Raw Images — (PDF) [file pbio.3002690.s007.pdf]

1B

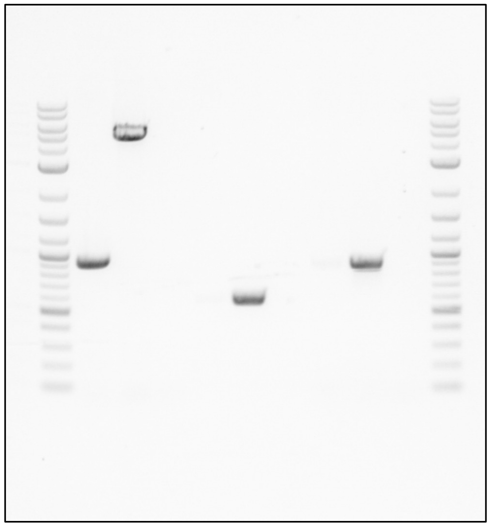

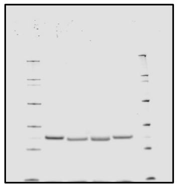

1C SKP1 Blot. (note that lanes 3-4 were samples not shown in the published figure

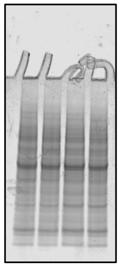

1C Commassie Stain Blot. (note that lanes 3-4 were samples not shown in the published figure)

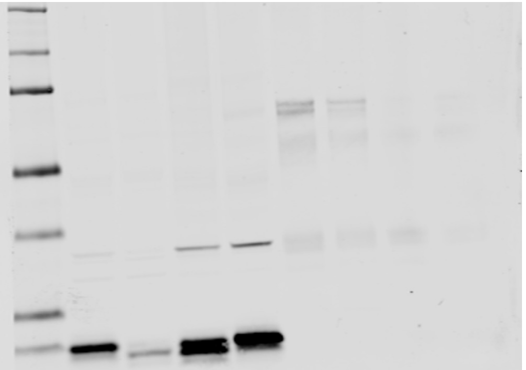

7A SKP1 Blot. (note that lanes 1-4 were samples shown in the published figure. Lanes 4-8 were the IP samples but no Skp1 signal detected since SKP1 and PHYa cannot be detected in an IP complex.

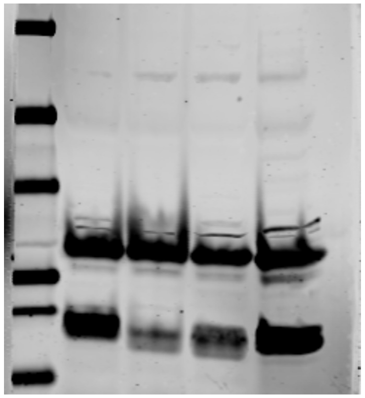

7A SAG1 Blot. (note that duplicate samples of lanes 1-4 from the above gel were run on this gel)

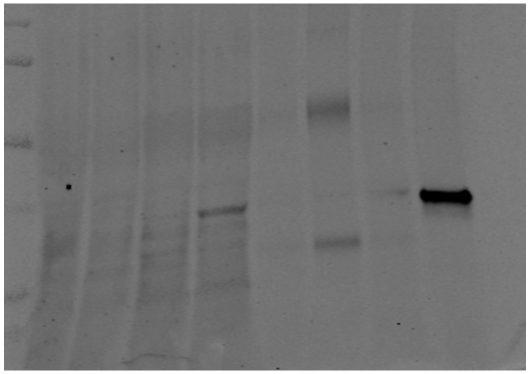

7A HA Blot. (note that lanes 1-4 were samples shown WCL lanes and 4-8 were samples for the IP blot.

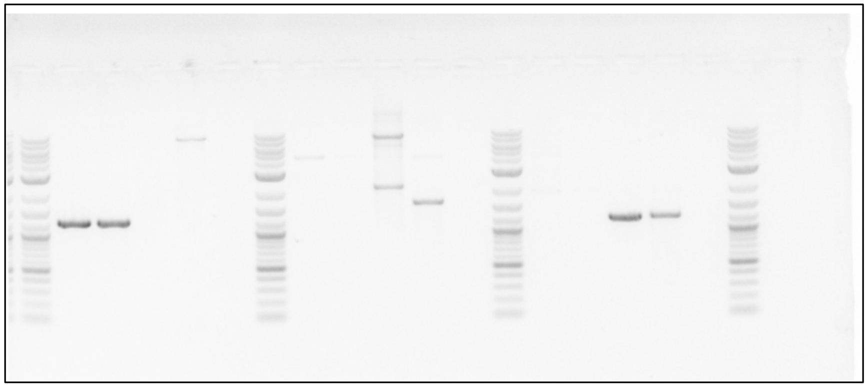

S3B DNA Gels
